# Genomic and Clinical Analysis of a Fatal Human *Lyssavirus irkut* Case: Evidence for a Natural Focus in the Russian Far East

**DOI:** 10.3390/v17060769

**Published:** 2025-05-28

**Authors:** Ekaterina Klyuchnikova, Anna Gladkikh, Olga Iunikhina, Valeriya Sbarzaglia, Elena Drobot, Margarita Popova, Irina Lyapun, Tatiana Arbuzova, Irina Galkina, Alena Sharova, Svetlana Abramova, Nadezhda Tsyganova, Eva Pugacheva, Edward Ramsay, Elena Poleshchuk, Larisa Somova, Daria Tagakova, Dmitry Pankratov, Gennady Sidorov, Nikolay Rudakov, Vladimir Dedkov, Mikhail Shchelkanov

**Affiliations:** 1 Saint Petersburg Pasteur Institute, 197101 Saint Petersburg, Russia; 2 G. P. Somov Institute of Epidemiology and Microbiology, 690087 Vladivostok, Russia; 3 School of Medicine and Life Sciences, Far Eastern Federal University, 690922 Vladivostok, Russia; 4 Omsk Research Institute of Natural Foci Infections, 644080 Omsk, Russia; 5 Faculty of Medicine and Prevention, Omsk State Medical University, 644099 Omsk, Russia; 6 Faculty of Natural Science Education, Omsk State Pedagogical University, 644099 Omsk, Russia; 7 E. I. Martsinovsky Institute of Medical Parasitology, Tropical, and Vector Borne Diseases, Sechenov First Moscow State Medical University, 119992 Moscow, Russia; 8 Federal Scientific Center of East Asia Terrestrial Biodiversity, 690022 Vladivostok, Russia

**Keywords:** lyssaviruses, *Lyssavirus irkut*, irkut virus, IRKV, rabies, antigenic site, bats, natural focus, Sikhote-Alin, primorsky krai

## Abstract

In this report, we document and analyze a case in which the Irkut virus (IRKV) (Mononegavirales: Rhabdoviridae) caused a fatal human case following a bat bite in June 2021. Unfortunately, the available data did not permit a detailed taxonomic classification of the carrier bat (Chiroptera). The event occurred in the southwestern part of the Sikhote-Alin mountain region (Russian Far East) covered by the Ussuri taiga forest. The symptoms of the illness began with the following: fever; pronounced psychomotor and motor agitation; tremor of the lower jaw and tongue; aphasia; dyslexia; and dysphagia. These rapidly developed, leading to a severe and fatal encephalitis. The patient was not vaccinated for rabies and did not receive rabies immunoglobulin. Using brain sections prepared from the deceased, molecular diagnostics were performed: immunofluorescence (polyclonal anti-rabies immunoglobulin) indicating the presence of the lyssavirus antigen; and RT-PCR indicating traces of viral RNA. Sectional material (brain) was used for whole-genome sequencing, resulting in a near-complete sequence of the lyssavirus genome. The obtained genomic sequence was identified as the Irkut virus. A comparative analysis of the new sequence and other currently available IRKV sequences (NCBI) revealed differences. Specifically, amino acid differences between antigenic sites in the isolate and those of the rabies vaccine strain used regionally were noted. The patient history and subsequent analysis confirm human IRKV infection following bat contact. Like other fatal cases of IRKV infection described earlier, this case occurred in the southern part of the Russian Far East. Two have occurred in the southwestern part of the Sikhote-Alin mountain region. This indicates the possible existence of an active, natural viral focus.

## 1. Introduction

Bats (Chiroptera) feature a number of amazing evolutionary adaptations, such as being the only mammals capable of true powered flight [1]. They are known to be a natural reservoir of a variety of pathogenic viruses. As such, potential human contact with bats represents a serious hazard [2,3]. Among the various bat viruses, there is a group of viruses belonging to the *Lyssavirus* genus (Mononegavirales: Rhabdoviridae) that are known as zoonotic neurotropic pathogens causing rabies, a severe fatal encephalitis in warm-blooded animals that can be transmitted to humans [2,4].

According to the current ICTV report, the genus *Lyssavirus* includes 17 species. Based on their antigenic properties and phylogenetic relationships, two phylogenetic (antigenic) groups of lyssaviruses are distinguished. Phylogroup I includes *L. rabies* (RABV—Rabies virus), *L. aravan* (ARAV—Aravan virus), *L. australis* (ABLV—Australian bat lyssavirus), *L. bokeloh* (BBLV—Bokeloh bat lyssavirus), *L. duvenhage* (DUVV—Duvenhage virus), *L. formosa* (TWBLV—Taiwan bat lyssavirus), *L. gannoruwa* (GBLV—Gannoruwa bat lyssavirus), *L. hamburg* (EBLV-1—European bat lyssavirus 1), *L. helsinki* (EBLV-2—European bat lyssavirus 2), *L. irkut* (IRKV—Irkut virus), and *L. khujand* (KHUV—Khujand virus). The second group (phylogroup II) includes *L. lagos* (LBV—Lagos bat virus), *L. mokola* (MOKV—Mokola virus), and *L. shimoni* (SHIBV—Shimoni bat virus). Three more lyssaviruses are not included in the groups described: *L. caucasicus* (WCBV—West Caucasian bat virus); *L. ikoma* (IKOV—Ikoma virus); and *L. lleida* (LLEBV—Lleida bat lyssavirus). Moreover, there are several related, unclassified viruses: Divaea bat lyssavirus; Kotalahti bat lyssavirus; Phala bat lyssavirus; and Taiwan bat lyssavirus 2 [5].

Lyssavirus genomes are negative-sense-RNA-encoded, typically ranging from 11,900 to 12,278 nucleotides. IRKV is known to have a genomic organization similar to other lyssaviruses, including structural protein genes for their respective proteins: nucleoprotein (N); phosphoprotein (P); matrix protein (M); glycoprotein (G); and the viral RNA-dependent RNA polymerase (L). In all lyssaviruses, these five structural genes are arranged in the order 3′-N-P-M-G-L-5′ [6]. Among the different viral species, some gene sites are similar in structure and length (N, M, and L), while others (P and G) are quite variable [6,7].

Lyssaviruses are distributed on all continents except Antarctica, while the geographical distribution of different lyssavirus species is variable. Currently, 15 of the 17 known lyssavirus natural reservoirs are Chiroptera. However, carnivores are also involved in their circulation [8]. Lyssaviruses are transmitted through the bites, scratches, and saliva of infected animals, or exposure to mucous membranes [8]. Currently, there is no evidence that arthropods are involved in lyssavirus transmission.

The most known representative lyssavirus is the widespread RABV. It causes about 99% of human rabies cases all over the world, and more than 55,000 human deaths per year, including those in developing countries [9,10]. In comparison to RABV, the risk of human infection with other lyssaviruses from bats appears to be quite low [11,12]. Few confirmed cases of human deaths from lyssaviruses as a result of bat bites have been established [13,14,15,16]. According to the information provided by the Russian Center for Hygiene and Epidemiology, requests for medical assistance after animal bites and scratches (both wild and domestic) exceeded 330,000 (annually) from 2016 to 2023. However, rabies infections among humans have not exceeded seven cases per year in Russia. The highest number of reported cases (7) was recorded in 2020. In 2021, there were six cases, including the one described here. In most cases, human rabies infection was associated with contact with wild or domestic predators (dogs, cats, wolves, foxes, raccoon dogs, etc.).

The first strain of IRKV was found in Russia from a great, or Siberian, tube-nosed bat (*Murina hilgendorfi*; outdated name—*Murina leucogaster*) captured in Irkutsk (Russia, Irkutsk region) in September 2002. The isolated lyssavirus strain was named and characterized: *L. irkut*/Murina leucogaster/Russia/IBS/2002 (GenBank ID EF614260), or *L. irkut*/Ref [17].

In 2007, the first lethal case of human infection with IRKV was described in detail: lyssavirus infection was confirmed in a woman living in the village of Ozernoe (Russia, Primorsky Krai, Yakovlevsky District) [18]; the *L. irkut*/Murina leucogaster/Russia/Ozernoe/2007 strain (GenBank ID NC_025408), or *L. irkut*/Ozernoe, was isolated from autopsy material of the deceased.

Three more cases of human death from IRKV were recorded in 2019–2021 in the Russian Far East: in the suburbs of Blagoveshchensk (Amur region), a 36-year-old man (August 2019); in the city of Fokino (Primorsky Territory), a 73-year-old man (August 2019); and in the Chuguevsky district (Primorsky Territory), a 35-year-old man. In all described cases, people did not seek medical attention before the first symptoms occurred. Isolated lyssaviruses were obtained from patient tissues post-mortem and were characterized as IRKV by fragments of the nucleoprotein gene (GenBank ID: OQ377548- OQ377550) [19].

IRKV strains are also known from Northeast China, such as the following: *L. irkut*/Murina leucogaster/China/Jilin-THChina12/2012 (GenBank ID JX442979); *L. irkut*/THChina12; *L. irkut*/dog/China/Jilin-FX17/2017 (GenBank ID MF737385); and *L. irkut*/FX17 [20].

The current study provides a detailed overview of the last fatal case (August 2021) associated with IRKV. In this work, we carry out a comparative analysis of the isolated IRKV with previously known sequences, and consider their phylogenetic relationships. The true burden of IRKV is that its prevalence among bats and its danger to humans are not currently clear. Thus, the aims of this work were as follows: to detail the clinical features of a lethal encephalitis case registered in 2021 (Primorsky Krai); to evaluate pathomorphological changes caused in infected model animals; to obtain the whole-genome sequence of IRKV; to characterize its organization; and to determine the phylogenetic relationships between the isolated IRKV and other lyssaviruses based on whole-genome analysis.

## 2. Materials and Methods

Ethics approval and consent to participate: The authors confirm compliance with institutional and national standards for the use of laboratory animals in accordance with Consensus Author Guidelines for Animal Use (IAVES, 23 July 2010). The study was conducted with the informed consent of patient legal representatives. The research protocol was approved by the following: the Ethics Committee of the Omsk Research Institute of Natural Focal Infections (protocol without number dated 27 January 2021); the local Ethics Committee of the St. Petersburg Pasteur Institute (St. Petersburg, Russia, No. 063-03); and the Somov Institute of Epidemiology and Microbiology in accordance with accepted recommendations for maintenance, euthanasia, and therapy in accordance with protocol No. 3 (dated 10/01/2021).

Isolation of the virus was carried out in three-week-old outbred white mice weighing 8–12 g by intramuscular injection of 0.03 mL of a 10% suspension of the brain of the deceased individual prepared in Hank’s solution with gentamicin [21,22,23]. Animals of similar age and weight were used as a control group and were intramuscularly injected with Hank’s solution with an antibiotic. According to standard virological guidelines, a control group of animals was used [22,23].

Three-week-old outbred white mice obtained from a certified laboratory animal nursery were used in the experiments. All animals were kept under standard conditions in accordance with Directive 2010/63/EU of the European Parliament and the Council of 22 September 2010 on the protection of animals used for scientific purposes [24] and Guidelines for the Care and Use of Laboratory Animals [25]. The light regime was 12 h during the day and 12 h in the dark. The mice received complete pellet feed and drinking water ad libitum. Animals were kept in standard plastic cages with no more than five individuals. In total, ten animals were used (five in the experimental group and five in the control group).

After intramuscular injection with 10% brain suspension from the deceased individual (2021), mice were monitored daily for 28 days, with symptoms of infection developing every 6–12 h. The specific symptom of rabies in vivo, hind limb paralysis, was adopted as the end point symptom for calculating survival time. Animals were euthanized after reaching the end point of the experiment by slow exposure to 100% CO_2_ at a flow rate displacing 10–30% of the cage volume/minute, followed by bleeding of the heart cavities. Viral infectious activity at the 3rd passage was determined by intramuscular injection of 3-week-old mice with a brain suspension 10-fold dilution in Hanks’ solution (10^−1^ to 10^−7^). Viral titer was calculated according to the method of Reed and Munch [26].

Microscopy was carried out on the brains of infected animals after euthanasia, which were primarily fixed for 1 h in a buffered solution (pH 7.2–7.4) of 2% glutaraldehyde. After 1 h, a part of the brain in the region of Amon’s horn was excised, chopped into pieces (1 × 1 mm), and left for further fixation (4 h). The fixed material was embedded in LR white acrylic resin epoxy (Sigma-Aldrich, Saint Louis, MO, USA) according to manufacturer instructions for light and electron microscopy. Brain samples embedded in resin were cut using an LKB-V Prodactor ultratome (LKB Inc., Stockholm-Broma 1, Sweden) into semi-thin sections up to 1 µm thick and stained with a multicolor dye based of methylene blue-azure II and basic fuchsin without removing the resin [27].

Total RNA for real-time RT-PCR was isolated from primary pathological material and the brain of infected mice using the Ribo-Zol-A kit^®^ (AmpliSens, Moscow, Russia). Real-time RT-PCR was performed with reagent kits for detection of rabies virus RNA OM-Rabies-RV (Syntol LLC, Moscow, Russia).

Total RNA isolation for library preparation (from brain tissue taken from the deceased individual) was obtained by extraction and purification using the QIAamp^®^ Viral RNA Extraction Kit^®^ (Qiagen, Hilden, Germany) with the QIAcube Connect automatic station (Qiagen, Hilden, Germany) according to manufacturer’s recommendations. RNA was eluted with 50 µL of AVE Buffer^®^ (Qiagen, Hilden, Germany) and stored at −70 °C until further analysis.

Library preparation and sequencing: Total isolated RNA was used for library preparation with KAPA RNA HyperPrep Kit (Kapa Biosystems Pty, Cape Town South Africa) according to manufacturer recommendations. For fragmentation and priming reaction, 10 ng of purified RNA was taken. The resulting libraries’ median lengths were 240–300 bp. The libraries were processed using V3 chemistry with 150 bp paired-end sequencing on Illumina MiSeq instrument.

Bioinformatic analysis of the quality of Illumina reads was assessed using the FastQC program [28]. Raw reads were filtered and trimmed with Trimmomatic (PE mode, ver. 0.39 USADELLAB) [29] with the following parameters: ILLUMINACLIP:TruSeq3-PE.fa:2:30:10 SLIDINGWINDOW:4:20 MINLEN:50. Trimmed reads were assembled with SPAdes. BLAST analysis was carried out against the RefSeq viral database in order to identify viral species. After virus identification, reads were secondary-mapped to *L. irkut*/THChina12 nucleotide sequence (GenBank ID JX442979) as reference genome with bowtie2 (v.2.3.5.1) [30] in local alignment mode. All reads were then assigned to read groups by Picard Toolkit (ver. 2.27.4, Broad Institute). The samtools and bcftools v. 1.18 software were used to generate consensus [31]. The complete genome sequence of *L. irkut*/FE-681 has been deposited with the international molecular genetic database under the GenBank ID OP616745.

Phylogenetic trees were constructed based on the genomic sequences of lyssavirus members using the maximum likelihood method implemented in MEGA12 [32]. The phylogeny was inferred using the Maximum Likelihood method and General Time Reversible model of nucleotide substitutions, and the tree with the highest log likelihood is shown. The percentage of replicate trees in which the associated taxa clustered together (1000 replicates) is shown in the nodes. The initial tree for the heuristic search was selected by choosing the tree with the superior log-likelihood between a Neighbor-Joining (NJ) tree and a Maximum Parsimony (MP) tree. The NJ tree was generated using a matrix of pairwise distances computed using the General Time Reversible model.

## 3. Case Description

On 9 August 2021, a previously healthy 35-year-old man was admitted to a regional infectious disease hospital in Vladivostok (Primorsky Krai, Russia). According to the patient, the disease manifested acutely on August 5 with symptoms that included fever and an increase in body temperature up to 38.6 °C. The patient treated his fever symptomatically with paracetamol. However, his symptoms persisted, and hyperthermia did not resolve. Upon examination, the patient revealed pronounced psychomotor and motor arousal, tremor of the lower jaw and tongue, aphasia, dyslexia, and dysphagia. In the Romberg pose, the patient was unstable. At the same time, consciousness remained clear. The blood pressure was 156/99 mm Hg, whereas the heart beat was 118 per minute. A computer tomography scan of the brain revealed no signs of pathological changes in brain tissue.

Nevertheless, in order to exclude infectious encephalitis, the patient’s blood was collected and studied for SARS-CoV-2 (severe acute respiratory syndrome coronavirus 2) (Nidovirales: Coronaviridae), *Orthoflavivirus encephalitidis* (TBEV—Tick-borne encephalitis virus) (Amarillovirales: Flaviviridae), *Simplexvirus humanalpha* {1, 2} (HSV-{1, 2}—Herpes simplex virus {1, 2}, or Human alphaherpesvirus {1, 2}) (Herpevirales: Herpesviridae), *Cytomegalovirus humanbeta 5* (HCMV—Human cytomegalovirus) (Herpesvirales, Orthoherpesviridae), *Mamastrovirus* sp. (HAstV—Human astroviruses) (Stellavirales: Astroviridae), *Enterovirus* sp. (EV—Enteroviruses) (Picornavirales: Picornaviridae), *Mycobacterium tuberculosis*, and *M. bovis* (Mycobacteriales: Mycobacteriaceae) using real-time RT-PCR/PCR. The results of the clinical blood studies are presented in Table 1.

Considering the severity of the patient’s condition, he was placed in an intensive care unit. Tracheal intubation was performed, and mechanical ventilation was connected. He was treated with the following: ceftriaxone (2.0 intravenously, t.i.d.); acyclovir (500 mg intravenously t.i.d.); cytoflavin (10 mL intravenously, droplet, b.i.d.); elzepam (1 mg/mL, 1 mL intravenously, b.i.d.); NaCl sol. 0.9% (250 mL intravenously, droplet, t.i.d.); dexamethasone (4 mg/mL intravenously, b.i.d.); propofol (20 mg/mL, 50 mL intravenously t.i.d.); fraxiparine (9500 ME, 0.6 mL intravenously, droplet, b.i.d.); and paracetamol (10 mg/mL, 100 mL intravenously, droplet, b.i.d.). Despite the efforts made, the patient’s condition continued to deteriorate. Generalized tremor, opsoclonus, bulbar syndrome, myoclonic eyelid retraction, and tetraparesis appeared. However, signs typical of the classical rabies, such as hydrophobia and photophobia, were not observed.

**Figure 1:**
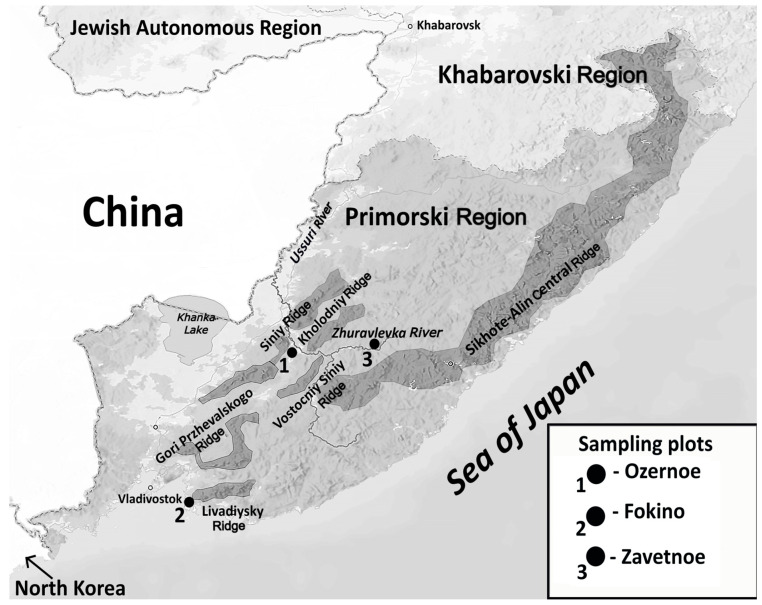
Geographical overview of the Sikhote-Alin natural foci of IRKV. The locations of human fatalities associated with IRKV in Primorsky Krai (Russian Far East), with indication of ridges in the southwestern part of the Sikhote-Alin mountain region, are indicated: case 1 (May 2019); case 2 (August 2019); and case 3 (June 2021), the current case.

On 12 August, during a survey of the patient’s relatives, information was received that, in mid-June 2021, he was bitten on the upper lip by a bat of unidentified species. The incident occurred in the Ussuri taiga forest, on the bank of the Zhuravlevka River flowing along the southwestern slope of the Central ridge of the Sikhote-Alin mountain region, in the vicinity of the village Zavetnoe (44°42′34″ N; 134°41′50″ E; 276 m above mean sea level) in the Chuguevsky District of Primorsky Krai, Russia (Figure 1). Cases of IRKV infection in humans have already been identified in this natural area. The locations of lethal human cases associated with IRKV in Primorsky Krai are shown in Figure 1.

Taking into account the epidemiological history, the patient was suspected to have acute encephalitis of lyssavirus etiology. The patient’s condition became critical and, on the eighth day of hospitalization after 30 min of unsuccessful resuscitation, a fatal outcome occurred. Autopsy material (brain tissue) was directed to the Reference Center for Rabies Monitoring (Omsk Institute of Natural Focal Infections, Rospotrebnadzor) for expert diagnostics. Antigen presence was confirmed by a fluorescent antibody test (FAT), and lyssavirus RNA was detected by PCR. A lyssavirus was isolated from an obtained autopsy sample and used to sequence the nucleoprotein gene [19]. The obtained nucleoprotein sequence was uploaded to NCBI (OQ377550).

Additionally, autopsy material was sent to specialists at Federal Service for the Oversight of Consumer Protection and Welfare (Rospotrebnadzor) centers for subsequent whole-genome sequencing, specifically the Saint Petersburg Pasteur Institute (St. Petersburg) and the G.P. Somov Institute of Epidemiology and Microbiology (Vladivostok). Viral genome L. irkut/FE681 was obtained from the autopsy material. A BLAST analysis revealed a more than 99% homology with the IRKV/L. irkut/THChina12 (JX442979) strain. Thus, conclusive evidence was obtained that the fatal acute encephalitis case was caused by the Irkut virus.

## 4. Results

To obtain samples for the microscopic characterization of pathological changes, necropsy material was obtained using a mouse model of infection (intramuscular inoculation in three-week-old mice). The symptoms led to death or euthanasia (8–12 days after infection) within 12–24 h in the context of lightning-fast clinical symptoms. These included the refusal of water and food, body weight loss, general tremor, swelling of the eyeballs, tousled hair, progressive paralysis of the hind limbs, and agony. The mortality rate was 100%. In the control group, mice remained healthy until the 28th day of observation. Out of five animals in the experimental group, three died from rabies disease, and two were euthanized 12 h after the development of specific clinical symptoms. The infectious viral titer at the third passage was 6.1 lg LD50, and the incubation period was reduced to 8–9 days.

Lyssavirus RNA was detected by RT-PCR as described earlier [19] in three various samples: autopsy material from the human case described; necropsy brain samples from infected mice after the first passage; and necropsy brain samples (infected mice) after the third passage. The Ct values were 23.5, 26.0, and 22.6, respectively.

**Figure 2:**
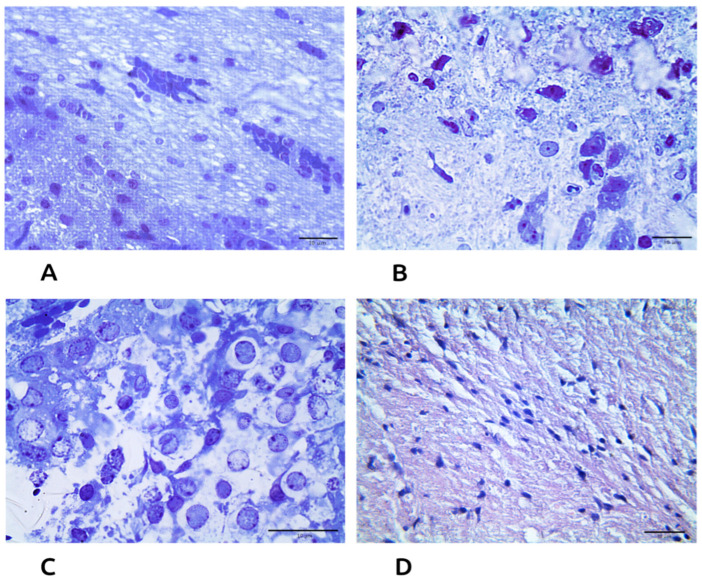
Histological changes in brain tissue of bioassay (*L. irkut*/FE-681) mice (methylene blue-azure II-basic fuchsin staining): (**A**) violation of cerebral circulation, magnification 600×, bar = 10 µm; (**B**) signs of encephalitis, magnification 600×, bar = 10 µm; (**C**) neuronal damage, magnification 1000×, bar = 10 µm; and (**D**) control, magnification 600×, bar = 10 µm.

Microscopy of the brains of experimentally infected mice revealed a wide range of structural changes. In parts of the capillary network, there were cases of plethora, pronounced pericapillary edema, aggregation of erythrocytes, marginal standing of leukocytes, edema, and stratification of the vascular wall, which indicated a violation of cerebral circulation (Figure 2A). *L. irkut*/FE-681 induced pathological changes in nervous tissue cells, neurophagia with the formation of the so-called glial nodules of rabies (Figure 2B). Neuronal damage was manifested by chromatolysis, hydropia, nuclear pyknosis, and necrosis. Hyperchromic and hypochromic neurons were identified (Figure 2C,D).

## 5. Genomic and Phylogenetic Analysis

Viral RNA was isolated from the brain tissue of a rabies-infected person (post-mortem). After sequencing and assembly, the resulting 11,979 bp viral sequence was identified as *L. irkut* and named *L. irkut*/FE-681. The obtained sequence was compared to the genomes of lyssaviruses available in NCBI. The highest nucleotide similarity (99.1%) was found with *L. irkut*/THChina12 [20]. With *L. irkut/*Ozernoe [18], the similarity was 98.7%. A comparison of the *L. irkut*/FE-681 nucleotide sequence obtained with those available in NCBI showed that the new virus differs from *L. irkut*/THChina12 by 110 substitutions; and it differs from *L. irkut*/Ozernoe by 160 substitutions. The similarities to different species of the phylogroup I of lyssaviruses are presented in Table 2.

In general, the Irkut viruses are highly similar, but IRKV/*L. irkut*/Ref differs quite significantly in both the nucleotide and amino acid sequence. *L. irkut*/FE-681 is the closest to the Chinese strain *L. irkut*/THChina12. An amino acid deletion at position 1352 in protein L was detected. The V1352 deletion probably has no effect on enzymatic activity, as it is not in a domain associated with such activity. It is located at the junction of the capping domain (CAP) and the connector domain (CD). A comparison of the amino acid substitution patterns of known IRKV are shown in Supplementary Materials.

When comparing *L. irkut*/FE-681 with the vaccine strain *L. rabies/*Vnukovo-32 (GeneBank ID OP642459) in terms of the G protein, a 68% similarity by nucleotide and 70% by amino acid sequence were noted. Amino acid sequence differences at G-protein-antigenic sites are presented in Table 3 for *L. irkut*/FE-681 and a rabies vaccine strain (*L. rabies*/Vnukovo-32).

**Figure 3:**
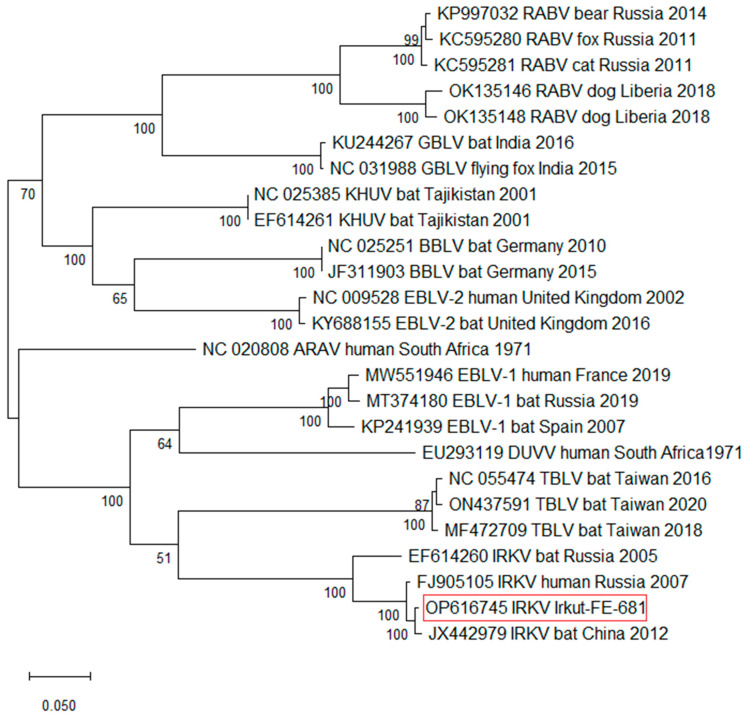
Maximum likelihood phylogenetic tree based on *Lyssavirus* complete genomic sequences. The reliability of the phylogeny was evaluated using bootstrapping with 1000 replicates.

A phylogenetic analysis confirms the similarity between the *L. irkut*/FE-681 and *L. irkut*/THChina12 strains, as seen by their clustering next to each other (Figure 3). All IRKV variants form a distinct group with maximal bootstrap support.

## 6. Discussion

Chiroptera is one of the most widely represented and taxonomically rich orders of mammals that have acquired, in the course of evolution, a number of physiological adaptations (primarily aimed at ensuring active flight and echolocation). This provides a high level of ecological plasticity and a wide geographical distribution of Chiroptera in almost all climatic zones, except the circumpolar ones [3,4]. It has been revealed that bats are the main natural reservoir for many RNA viruses associated with zoonotic diseases (including lyssaviruses), as well as those with pandemic potential [4]. The majority of lyssaviruses have a single host reservoir. The only exception was RABV associated with several species of bats and carnivores [33].

Representatives of eight bat genera are found in Primorsky Krai: Serotine bats (*Eptesicus*); Serotine-like pipistrelles (*Hypsugo*); Bent-winged bats (*Miniopterus*); Tube-nosed bats (*Murina*); Mouse-eared bats (*Myotis*); Pipistrelles (*Pipistrellus*); Long-eared bats (*Plecotus*); and Particolored bats (*Vespertilio*). Twelve species are distributed over almost the entire region: Northern bat (*Eptesicus nilssonii*); Alashanian pipistrelle (*Hypsugo alashanicus*); Hilgendorf’s tube-nosed bat (*Murina hilgendorfi*); Ussurian tube-nosed bat (*Murina ussuriensis*); Bombinus bat (*Myotis bombinus*); Ikonnikov’s myotis (*Myotis ikonnikovi*); Long-tailed myotis (*Myotis longicaudatus*); Eastern water bat (*Myotis petax*); Siberian bat (*Myotis sibiricus*); Ognev’s long-eared bat (*Pipistrellus ognevi*); Particolored bat (*Vespertilio murinus*); and Asian particolored bat (*Vespertilio sinensis*). Some species, such as the Eastern bent-winged bat (*Miniopterus fuliginosus)*, big-footed bat (*Myotis macrodactylus*), and Japanese house bat (*Pipistrellus abramus)*, are found in the extreme southern part of the region, with the majority of their range being much further south [1,34].

As for IRKV, it has been found in only the species *Murina leucogaster* (modern name—*Murina hilgendorfi*) [19,20]. According to observations, Hilgendorf’s tube-nosed bat, Northern bat, and Ognev’s bat are common and numerous bat species exist in southern parts of Eastern Siberia and the Russian Far East.

Taking into account previous cases of human infection with IRKV, it is possible to assume the existence of a natural focus of IRKV in the southwestern spurs of the Sikhote-Alin. In the reported human cases of lyssavirus infections following bat bites, the bat species have not been identified [19]. However, results from studies of IRKV in Hilgendorf’s tube-nosed bat reported in the literature suggest that it is highly likely that *Murina hilgendorfi* may be the primary host of IRKV, although other bat species should also be examined for IRKV [20]. Moreover, there is information about the IRKV infection of other animal species, such as dogs [35]. During the XXI century, all cases of human infection with IRKV took place in the Russian Far East and in the northeastern provinces of China [19,20].

The large tube-nosed bat (*Murina leucogaster*) inhabits East Asia, including China, Korea, Japan, Mongolia, and eastern Russia. Hilgendorf’s tube-nosed bat has been documented across multiple mountain ridges in the Sikhote-Alin range, which is characterized by a monsoon climate and Ussurian taiga forests landscapes. Further ecological and virological studies are necessary in order to monitor IRKV and other lyssavirus infections in bat populations. The clarification of the boundaries of viral circulation, and the better identification of pathogen carriers, are also needed. We assume the existence of a natural focus in Sikhote-Alin. However, the situation in the central and northern regions of Primorsky Krai remains unclear, and the margins of the focus may be wider.

Like other lyssaviruses, IRKV causes fatal neurological disease that is clinically and pathologically indistinguishable from symptoms caused by RABV. The disease develops as meningoencephalitis complicated by hyperthermic syndrome (up to 39–40 °C), intoxication, convulsive syndrome, and impaired speech and consciousness, as well as severe cardiovascular, respiratory, and cerebral insufficiency [19]. The experimental infection of mice with the *L. irkut*/FE-681 isolate, and the examination of pathological changes in nerve cells, showed that IRKV causes typical damage to nerve cells similar to that caused by RABV (Figure 2 and Figure 3). The incubation period for lyssavirus infection varies depending on the site of injury. In Russia, described and documented cases of lyssavirus infection that were acquired via bat bites are rare. After the development of the initial symptoms, the disease develops rapidly within one to two weeks, leading to a fatal outcome [18,19].

This study details the third reported case of human disease associated with IRKV that has occurred in the Russian Far East. The incubation period corresponded to 52 days, while the incubation periods for other lyssaviruses vary from 20 to 90 days, with some even lasting years [36]. After the initial symptoms appeared, the patient died within 10 days. This course of disease corresponds to those described in the literature and established for other cases of IRKV-associated encephalitis. As in the other described cases, paresis and paralysis were observed. In experimental animals, these clinical signs develop in 80% of cases after the intramuscular injection of IRKV isolate [37].

Fluorescent antibody testing continues to be the indicated method for the detection of lyssavirus infection [38], but the method detects all lyssaviruses (including IRKV) without distinguishing between species. Sequencing can give insight into the species causing the disease. Such a genetic analysis supports studies aiming to understand the differences between species, and it may assist in medical prevention tactics [39,40].

Lyssaviruses evolve more slowly than other rhabdoviruses [41], forming a monophyletic group with phylogroups based on serological data [42]. While antibodies for detecting RABV work against phylogroup I lyssaviruses, they are ineffective against phylogroup II [43]. Rabies vaccines provide cross-neutralizing immunity against various phylogroup I viruses including EBLV-1 [43,44], ARAV, and EBLV-2 [45], though their effectiveness varies. Pre-exposure RABV vaccination offers significant, but incomplete, protection against IRKV, with reduced protection in a post-exposure model [46].

**Figure 4:**
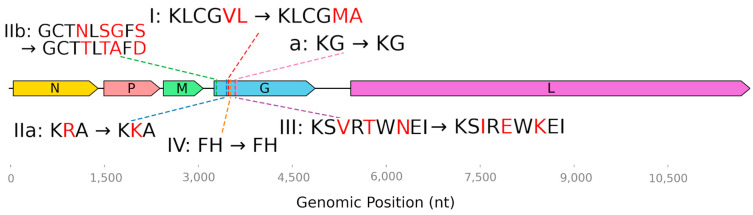
Amino acid substitutions at antigenic sites in the viral proteins of *L. irkut*/FE-681 relative to a vaccine strain (*L. rabies*/Vnukovo-32).

Taking these experiments into account, we decided to check the correspondence of the antigenic sites of the Irkut lyssavirus to the rabies strain that is used to create all rabies vaccines produced in the Russian Federation. Some amino acids located in immune determinant sites were found to be different (Figure 4).

Glycoprotein G is the target of all currently known neutralizing antibodies [47]. Four major antigenic sites (I, II, III, and IV) and a minor antigenic site “a” have been described [47]. For site I, the minimal binding region has been identified as KLCGVL (aa 226–231, numbering after the removal of the 19 aa signal peptide) [48]. In the *L. irkut*/FE-681 sequence, it is represented by KLCGMA. At the IIb antigenic site (aa 34–42), L. rabies/Vnukovo-32 contains GCTNLSEFS; and *L. irkut*/FE-681 features GCTTLTAFD. The substitution R199K takes place at the IIa site. Amino acid sequence differences were also found at antigenic site III (Table 3, Figure 4).

Similar data on the divergence of IRKV within phylogroup I have been previously described [49,50]. We take into account the limitations of the theoretical comparisons of amino acid sequences when evaluating the effectiveness of vaccination against the IRKV virus from phylogroup I. It is not possible to draw conclusions about the efficiency of pre- or post-exposure prophylaxis solely based on these comparisons insofar as all components of the immune response, including cell-mediated mechanisms, play an important role. Differences in the molecular structure between IRKV antigenic regions and those of rabies vaccine strains, including their role in the effectiveness of post-exposure immunotherapy, are open topics for discussion and further research. It can be assumed that the features of the IRKV antigenic site described here may influence the efficiency of rabies vaccines used against the Irkut virus. However, detailed experimental studies are needed before specific practical recommendations can be given. In addition, the paired cross-neutralization features of lyssaviruses need to be studied in detail.

## 7. Conclusions

The described cases of acute fatal encephalitis associated with IRKV following bat bites should be of concern due to the presence of an active, bat-associated natural focus of this virus in the southwestern part of Sikhote-Alin mountain region. This geography includes the southern tip of the Central ridge, Gori Przhevalskogo ridge, Livadiyskiy ridge, Vostochniy Siniy ridge, Kholodniy ridge, and the Siniy ridge. The Sikhote-Alin natural focal area requires further study in order to be able to draw conclusions about the activity of the IRKV natural focus. A further cause of concern should be the lack of reliable data about standard pre- and post-exposure vaccination against RABV in relation to potential effectiveness against (cross-protection from) IRKV. To avoid being bitten by bats, people should avoid approaching them or causing any disturbance. Taking into account the established cases of human fatality after contact with bats, individuals who have been bitten by bats (or other unvaccinated animals) should be strongly advised to immediately receive post-exposure prophylaxis.

## Figures and Tables

**Table 1:** Laboratory blood test results for the patient with acute encephalitis *.

Clinical Indicator	Value for the Sampling Data			Norm
	09/08/2021	10/08/2021	12/08/2021	
WBC (white blood cells), 10^9^/L	8.9	**19.54**	**12.6**	4.0–9.0
RBC (red blood cells), 10^12^/L	4.9	4.98	5.2	3.5–5.5
HGB (hemoglobin), g/L	**166**	154	**163**	110–160
MCV (mean corpuscular volume), fL	93	87.6	86.6	80.0–100.0
HCT (hematocrit), %	45.2	43.6	45.1	37.0–54.0
MCHC (mean corpuscular hemoglobin concentration), g/L	**377**	353	**361**	320–360
MCH (mean corpuscular hemoglobin), pg	**34.2**	30.9	31.3	27.0–34.0
PLT (platelet), 10^9^/L	197	192	185	150–400
MPV (mean platelet volume), fL	11.1	11.0	10.8	6.5–12.0
P-LCR (platelet larger cell ratio), %	34.3	33.3	30.3	11.0–45.0
NEU (neutrophils), 10^9^/L	5.7	**14.61**	**10.5**	2.0–7.0
NEU (neutrophils), %	65	**74.7**	**82**	50–70
LYM (lymphocytes), 10^9^/L	1.7	1.4	1.25	0.8–4.0
LYM (lymphocytes), %	18.7	11.1	9.9	20.0–40.0
MON (monocytes), 10^9^/L	N/A	2.76	0.9	0.12–1.20
MON (monocytes), %	N/A	**14.1**	7.1	3.0–12.0
EO (eosinophils), 10^9^/L	N/A	0.0	0.01	0.02–0.5
EO (eosinophils), %	N/A	0.0	**0.1**	0.5–5.0
BASO (basophils),10^9^/L	N/A	0.01	0.02	0.00–0.10
BASO (basophils), %	N/A	0.1	**0.2**	0.0–1.0
MXD (monocytes–basophils–eosinophils mixed), 10^9^/L	**1.5**	N/A	N/A	0.09–0.6
MXD (monocytes–basophils–eosinophils), %	**16.3**	N/A	N/A	3.0–11.0
RDW-SD (red cell distribution width—standard deviation), fL	N/A	40.8	39.8	39.9–52.2
RDW-CDCV (red cell distribution width—coefficient of variation), %	N/A	13.1	12.7	12.2–14.6
PDW (platelet distribution width), fL	N/A	12.3	12.1	9.8–15.2
Na^+^, mM	**151.3**	**146.0**	144.0	136–145
K^+^, mM	**3.0**	3.5	3.9	3.5–5.1
RNA *SARS-CoV-2*, swab, RT-PCR	Negative	N/A	N/A	Negative
DNA *Mycobacterium tuberculosis/bovis*, CSF, PCR	N/A	N/A	Negative	Negative
DNA *Simplexvirus humanalpha* {1, 2}, CSF, PCR	N/A	N/A	Negative	Negative
DNA *Cytomegalovirus humanbeta 5*, CSF, PCR	N/A	N/A	Negative	Negative
RNA *Enterovirus* sp., CSF, RT-PCR	N/A	N/A	Negative	Negative
RNA *Orthoflavivirus encephalitidis*, CSF, RT-PCR	N/A	N/A	Negative	Negative
RNA *Mamastrovirus* sp., stool, RT-PCR	N/A	N/A	Negative	Negative

* Notes: values that exceeded the norm are highlighted in bold; N/A—not applicable.

**Table 2:** Similarity of the *L. irkut*/FE-681 genome with other selected representatives of phylogroup I lyssaviruses.

		Sequence Similarity (%)					
GenBank ID	*Lyssavirus* Species Member/Strain	Complete Genome, Nucleotide Similarity (%)	Gene (Nucleotide/Amino Acid)				
			N	P	M	G	L
EF614260	IRKV/*L. irkut*/Ref	91	92/99	91/95	92/99	92/98	91/100
JX442979	IRKV/*L. irkut*/THChina12	99.1	99/100	99/99	99/100	99/100	99/100
FJ905105	IRKV/*L. irkut*/Ozernoe	98.7	99/100	99/99	99/100	99/100	98/100
MW551946	EBLV-1/*L. hamburg*/01humFRA	74	79/92	71/70	81/94	66/80	76/96
MF472709	TWBLV/*L. formos*a/YL/2017	71	81/91	67/64	78/94	74/68	75/95
NC_025385	KHUV/*L. khujand*/Khujand	71	76/88	67/39	77/91	70/77	73/95
NC_020810	DUVV/*L. duvenhage*/86132SA	71	77/90	67/64	79/92	71/74	75/95
NC_025251	BBLV/*L. bokeloh*/21961	71	76/89	69/40	78/89	71/75	72/95
NC_020808	ARAV/*L. aravan*/Kyrgyzstan	71	76/90	65/39	77/93	71/77	73/95
NC_009528	EBLV-2/*L. helsinki*/RV1333	71	76/87	67/40	77/87	7176	73/94
KY688155	EBLV-2/*L. helsinki*/RV3370	71	76/87	67/40	77/87	71/76	73/94
NC_031988	GBLV/*L. gannoruwa*/RV3266	69	74/88	64/36	75/86	69/74	72/94
OK135146	RABV/*L. rabies*/18008LIB	69	75/87	64/36	76/87	68/70	71/94
OK135148	RABV/*L. rabies*/18018LIB	69	75/87	64/35	75/87	68/70	71/93
OP642459	RABV/*L. rabies*/Vnukovo-32	68	75/87	64/37	75/82	68/68	71/93

**Table 3:** Comparison of G-protein-antigenic sites in *L. irkut*/FE-681 and a vaccine strain (*L. rabies*/Vnukovo-32).

Virus/Strain	Antigenic site (Amino Acids)					
	I (226–231)	IIa (198–200)	IIb (34–42)	III (330–338)	IV (263–264)	a (342–343)
RABV (*L. rabies*/Vnukovo-32)	KLCGVL	KRA	GCTNLSGFS	KSVRTWNEI	FH	KG
IRKV (*L. irku*t/FE-681)	KLCG**MA**	K**K**A	GCT**T**L**TA**F**D**	KS**I**R**E**W**K**EI	FH	KG

## Data Availability

The original contributions presented in this study are included in the article/Supplementary Material. Further inquiries can be directed to the corresponding author.

## Supplementary Materials

The following supporting information can be downloaded at: https://www.mdpi.com/article/10.3390/v17060769/s1, Table S1: Comparison of amino acid substitution patterns of known IRKV.
